# Comprehensive analysis of expression profiles and prognosis of TRIM genes in human kidney clear cell carcinoma

**DOI:** 10.18632/aging.204102

**Published:** 2022-05-26

**Authors:** Junwen Shen, Rongjiang Wang, Yu Chen, Zhihai Fang, Jianer Tang, Jianxiang Yao, Jianguo Gao, Wenxia Zhou, Xiongnong Chen

**Affiliations:** 1 The Department of Urology, The First Hospital of Huzhou, Zhejiang Province, China; 2 The Department of Operation Management, The First Hospital of Huzhou, Zhejiang Province, China

**Keywords:** TRIM genes, kidney clear cell carcinoma, prognosis

## Abstract

Objective: To determine survival rates and the underlying mechanism of genes in the TRIM family in Kidney Clear Cell Carcinoma (KIRC).

Methods: Transcriptional and survival data of TRIM genes in KIRC patients were retrieved from the UCSC Xena, and GEPIA databases. The function of TRIM genes in KIRC was investigated, focusing on potential ubiquitination, miRNAs regulation, and enrichment analysis. Next, TRIM gene survival values were determined, followed by the development of a survival-related signature.

Results: Only TRIM26 was down expressed in the carcinoma tissue and had a survival value in KIRC relative to control tissues, which was supplied by vitro experiment. The patients with lower expression of TRIM26 would have the chance to live a shorter time. SNRPB, which also plays a role in ubiquitination, directly interacted with TRIM26. Moreover, two miRNAs (hsa-let-7i-5p, and hsa-miR-1228-5p) that regulated levels of TRIM26 expression were also identified. Next, we constructed a signature (TRIM4/7/27/58/65/72) and found that high-risk scores of the signature were associated with poor survival rates in KIRC patients. while its resultant risk scores were correlated with immune cell components and markers.

Conclusions: TRIM26 was differentially expressed between KIRC and normal tissues and had a survival value in the KIRC. hsa-let-7i-5p/hsa-miR-1228-5p-TRIM26-SNRPB was a potential mechanism axis that may play a role on the KIRC cells. A survival signature (TRIM4/7/27/58/65/72) was successfully established to predict the survival of KIRC patients.

## INTRODUCTION

The tripartite motif (TRIM) family is a conserved motif that contains specific moieties, including a Really Interesting New Gene (RING) domain, B-boxes and Coiled-Coil (CC) regions. The C-terminal region of TRIM proteins is more variable and can consist of a number of different protein-protein association domains. Development of these TRIM complexes creates a challenge when distinguishing the physiological role of each protein, necessitating elucidation of the primary enzymatic E3 ubiquitin ligase function of TRIM proteins that target specific substrates within the ubiquitin cycle [1]. Ubiquitination, a common post-translational modification, is generally associated with protein degradation [2]. Notably, ubiquitination mediates the interaction between TRIM genes with multiple other forms of regulation, such as protein transport, nuclear factor kappa-light-chain-enhancer of activated B cells (NF-κB) signaling, and chromatin remodeling [3].

Previous studies have focused on the relationship between various TRIM family proteins and several forms of cancer and found that TRIM proteins play key regulatory roles in tumor suppressor pathways, such as regulating p53 degradation, through direct binding with TRIM39 [4]. In addition, numerous TRIM family proteins have exhibited different expression patterns, with corresponding survival value in many cancer types. For example, TRIM15 was associated with cell migration and tumor growth in colon cancer [5], while loss of TRIM62 expression has been reported in multiple human cancers [6]. However, only a handful of studies have evaluated the role of TRIM family proteins in patients with Kidney Clear Cell Carcinoma (KIRC). In the present study, we hypothesized that TRIM family proteins may be playing an essential role in KIRC patients. Therefore, we evaluated survival rates and mechanism of genes in the TRIM family in relation to KIRC.

## MATERIALS AND METHODS

### Data sources

We searched the GeneBank [7] (https://www.ncbi.nlm.nih.gov/gene/) website and retrieved a list of TRIMs genes associated with the human population. RNA sequence (RNASeq) and clinical phenotypic datasets for TCGA Kidney Clear Cell Carcinoma (KIRC) were downloaded from the UCSC Xena database [8] (https://xenabrowser.net/datapages/).

### Differential gene expression analysis

We performed group gene expression and survival analyses on the UALCAN portal [9] (http://ualcan.path.uab.edu/). Next, we used UALACN to analyze expression patterns of TRIMs genes, then applied GEPIA [10] (http://gepia.cancer-pku.cn/), a web-based tool that facilitates expression and function analysis based on TCGA and GTEx data, to correlate expression levels and pathological stages of KIRC patients. Results of GEO datasets were presented using GraphPad Prism 8.0 software.

### Survival analysis

We used GEPIA and UALCAN to perform Kaplan Meier (KM) analysis on target genes from the KIRC datasets. Specifically, “survival” and “glmnt” packages, implemented in R, were used to compute time-dependent ROC curves and nomogram models. Thereafter, univariate and multivariate Cox proportional hazard regression analyses were applied to examine clinical information and target genes for survival value.

### Functional enrichment analysis

We compared KIRC patients with expression profiles of the top 50 highest or lowest TRIM26 as the TRIM26 relative genes, and identified those that were significantly differentially expressed using the limma package [11] in R (log FC > 1 or log FC <-1, and P <0.01). We selected hub genes from the differentially expressed ones, using the STRING database and visualized them using Cytoscape software [12]. These top 50 hub and TRIM genes were subsequently used for GO/KEGG enrichment analyses [13].

### Prediction of downstream genes regulating ubiquitination

Firstly, we used the iUUCD dataset [14] (http://iuucd.biocuckoo.org/), which annotates the key regulators in modulating ubiquitin and ubiquitin-like conjugations, to predict the direct interaction and physical association. Secondly, we searched ubibrowser [15] (http://ubibrowser.ncpsb.org.cn/v2/home/index), a website that analyzes ubiquitin ligase/deubiquitinase substrate interactions, to identify potential ubiquitin correlations. Next, we used the UALCAN portal to analyze expression levels of potential downstream genes and their survival value in the KIRC dataset. Finally, we employed the TIMER website [16] (http://timer.cistrome.org/) to correlate TRIM with potential downstream genes.

### Prediction and analysis of upstream miRNAs

To investigate miRNAs upstream of target TRIM genes, we employed four miRNA prediction databases (miRDB, miRWalk, RNA22, and TargetScan) [17–20], with emphasis on potential miRNAs supported by at least two miRNA prediction databases. Thereafter, we used UALCAN to detect expression of target miRNAs and their survival value in KIRC patients. Finally, we correlated expression of these miRNAs with that of TRIM genes.

### Construction and analysis of the risk- score-based signature

We applied LASSO and cox proportional hazard regression to construct a risk score signature, using “survival” and “glmnt” packages in R. Analyses were performed twice. Firstly, we focused on all TRIM genes, then TRIM26 as well as their relevant 20 hub genes. Next, we compared the prognostic value of two risk scores using a time-dependent ROC analysis. Finally, we selected the best performing risk scores and identified their clinical features as well as prognostic value in KIRC.

### Analysis of tumor-infiltrating immune cells

TIMER (http://timer.cistrome.org/) is a web server for comprehensive analysis of tumor-infiltrating immune cells. Currently, TIMER is used for estimation of function, which analyzes the immune cells with TIMER, CIBERSORT, quanTIseq, XCELL, MCP-counter, and EPIC methods. In the present study, we focused on CIBERSORT and XCELL results, and identified different immune cells between KIRC patients with high or low risk scores. These were subsequently stratified into two group, namely 50% with high- and 50% with low-risk scores. These immune cells were retrieved, then correlated with survival value. Finally, we explored the risk score and expression profiles of 8 key immune-related genes, namely LAG3, PDCD1, CTLA4, TIGIT, PDCD1LG2, CD274, HAVCR2, and SIGLEC15.

### Cell lines

The 293T cell line (a human embryonic kidney cell line), and caki-1 cell line (a human renal clear cell carcinoma cell line) were acquired from the Chinese national collection of authenticated cell cultures. These cell lines were used to assess TRIM levels between benign and malignant renal cells. Cell cultures were performed based on the guidelines of the Chinese national collection of authenticated cell cultures.

### Quantitative real-time polymerase chain reaction (Q-PCR) and western blot (WB) analyses

These analyses were performed to analyze TRIM gene expression levels between benign and malignant renal cells. Both assays were performed as previously described. Q-PCR [21] was performed using the TOYOBO ReverTra Ace Q-PCR RT system with TOYOBO PCR primers. In WB analysis [22], primary antibodies against β-actin and TRIM genes as well as secondary antibodies were purchased from Boiss (Beijing, China). β-actin was the internal reference. Western blot images were analyzed using the Image J software.

### Data availability statement

Original contributions presented in the study are included in the article. All the data was obtained from public databases.

### Ethics statement

Ethical review and approval were not required for the study in accordance with the local legislation and institutional requirements. Similarly, written informed consent was not required for participation in this study in accordance with the national legislation and the institutional requirements.

## RESULTS

**Figure 1:**
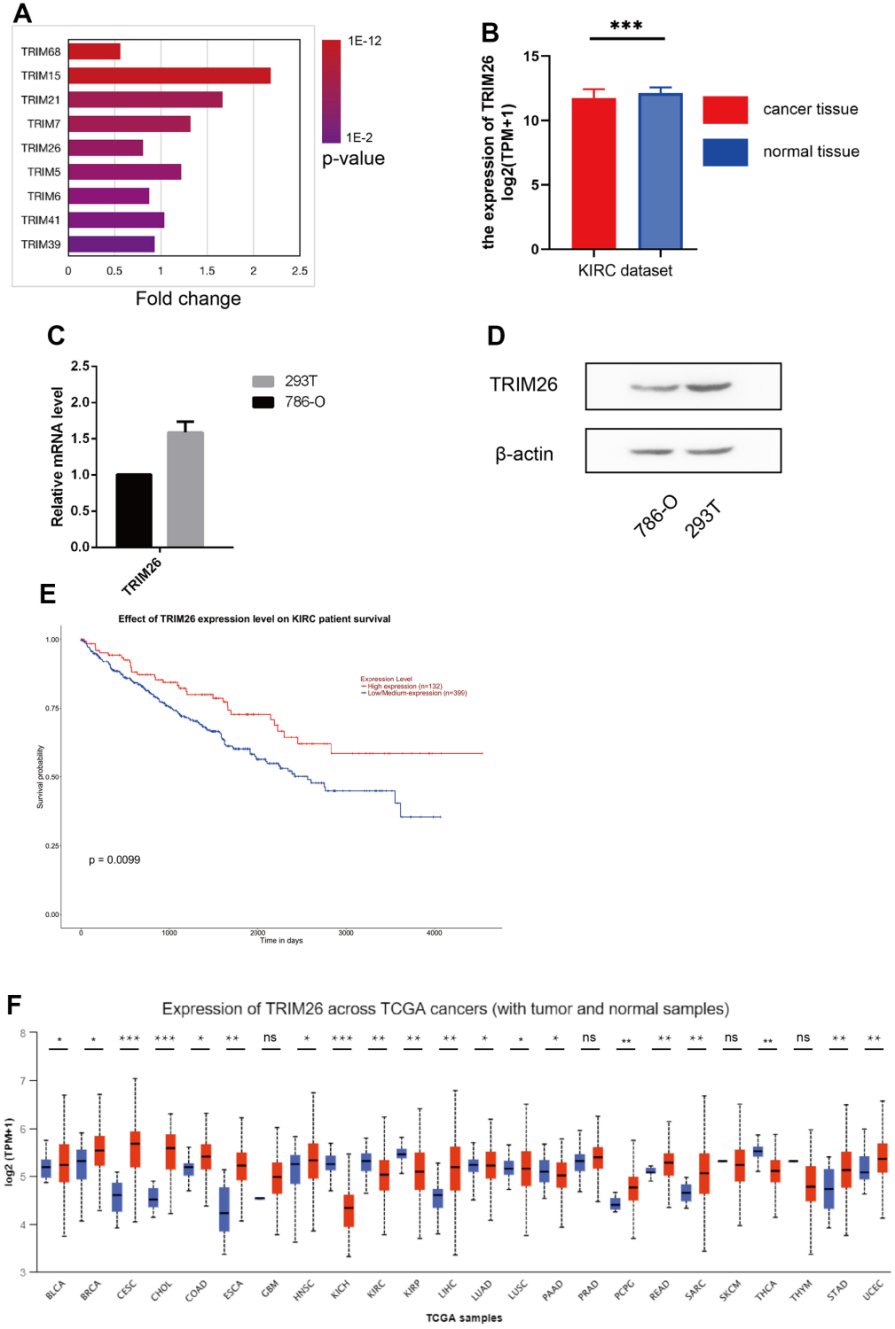
(**A**) 9 TRIM genes expression level in the KIRC dataset; (**B**) TRIM26 expression level in the KIRC dataset; (**C**, **D**) TRIM26 expression level in Q-PCR or WB analysis; (**E**) The survival analysis of TRIM26 in the KIRC dataset by the UALCAN; (**F**) The TRIM26 expression level in the TCGA pan-cancer tissue.

Analysis of sequence data revealed differential expression of target genes, between cancer and normal tissues in KIRC. Summarily, a total of 32 TRIM genes, namely TRIM1/4/5/6/7/10/11/15/17/21/22/25/26/27/34/35/38/39/41/43/47/48/50/53/58/60/62/64/65/68/69/72 were identified in human kidney cancer and normal tissue from the GeneBank. Expression analysis, using UALCAN on these genes in cancer vs. normal tissues, resulted in 9 differentially expressed TRIM genes (TRIM5/6/7/15/21/26/39/41/68) (P <0.001, Figure 1A, 1B). KM curves of the 9 TRIM genes revealed that only TRIM15 and TRIM26 had significant survival value for KIRC patients (P < 0.01, Figure 1E). In caki-1 cells, TRIM26 genes were down-regulated while TRIM15 did not exhibit significant variations when compared to 293T cells (Figure 1C). Moreover, WB analysis revealed differential trim26 protein levels between benign and malignant renal cells (Figure 1D). We also used the UALCAN pan-cancer view function to reveal expression patterns for TRIM26 across 24 TCGA cancer vs normal tissues (Figure 1F).

### Survival analysis and clinical information

**Figure 2:**
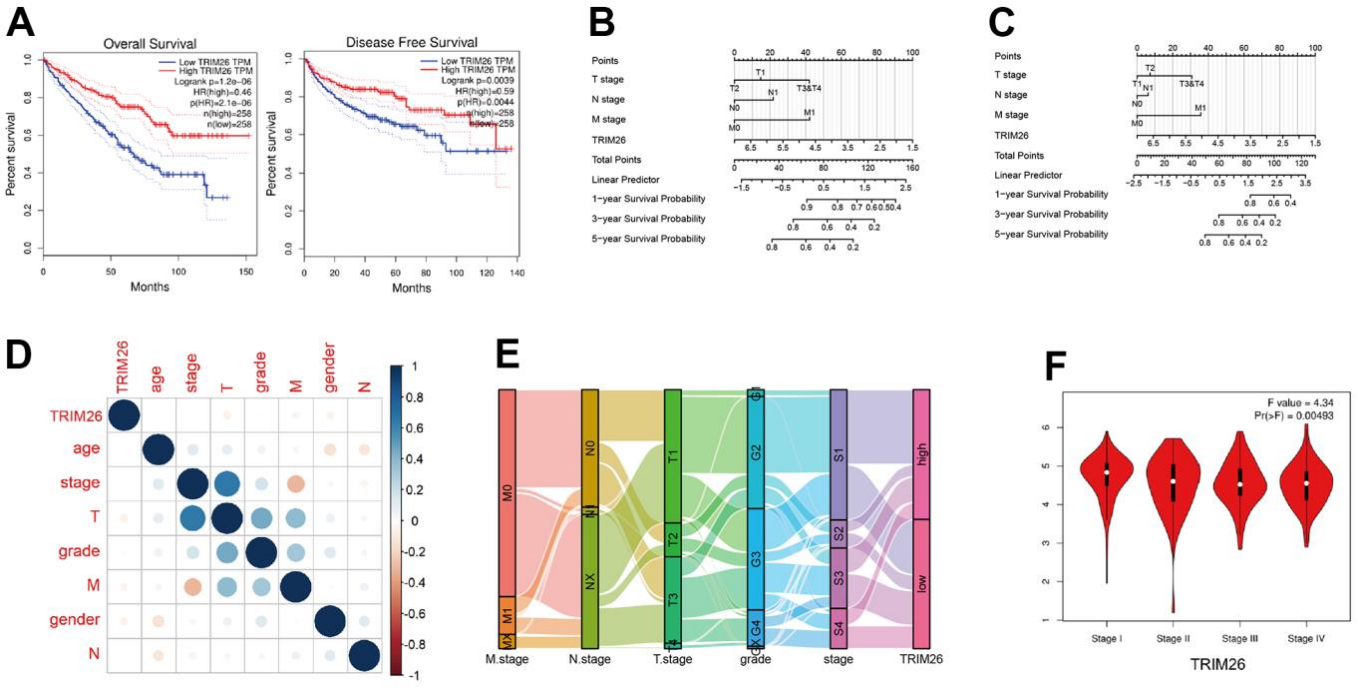
(**A**) The TRIM26 over survival(OS) time and disease free(DFF) survival time in the GEPIA; (**B**) The OS nomogram of TRIM26 for the KIRC patients; (**C**) The DFF nomogram of TRIM26 for the KIRC patients; (**D**) The correlation between TRIM26 expression and other clinical information; (**E**, **F**) The expression level of TRIEM26 in four pathological stages.

Firstly, we used the UALCAN and GEPIA to generate KM curves for TRIM26. Notably, GEPIA divided the KIRC patients into two equal groups. The results showed that KIRC patients with low expression of TRIM26 had worse overall and disease-free survival times (Figure 2A), relative to those with high expression (P <0.01). We used TRIM26 to establish a nomogram model for predicting overall survival and disease specific survival rates of patients (Figure 2B, 2C), then performed univariate and multivariate Cox proportional hazard regression. Results from univariate cox regression showed that T stage, N stage, M stage, and pathological stages, as well as age, and TRIM26 had significant survival value (P <0.001), while those from multivariate regression indicated that only M stage, age, and TRIM26 had survival value (P <0.01) (Table 1). Next, we correlated expression of TRIM genes with other clinical information in KIRC patients (Figure 2D) and found that most of KIRC patients with pathological stage 3 or stage 4 had significantly lower TRIM26 expression (Figure 2E). Moreover, TRIM26 was significantly differentially expressed across all pathological 4 stages (P =0.00493, Figure 2F).

**Table 1:** The basic clinical information of different expression TRIM15 and TRIM26 in KIRC data.

**Characteristic**	**Low expression of TRIM15**	**High expression of TRIM15**	**p**	**Low expression of TRIM26**	**High expression of TRIM26**	**p**
n	269	270		269	270	
T stage, n (%)			0.010			< 0.001
T1	122 (22.6%)	156 (28.9%)		110 (20.4%)	168 (31.2%)	
T2	38 (7.1%)	33 (6.1%)		39 (7.2%)	32 (5.9%)	
T3	100 (18.6%)	79 (14.7%)		111 (20.6%)	68 (12.6%)	
T4	9 (1.7%)	2 (0.4%)		9 (1.7%)	2 (0.4%)	
N stage, n (%)			0.011			0.759
N0	124 (48.2%)	117 (45.5%)		118 (45.9%)	123 (47.9%)	
N1	14 (5.4%)	2 (0.8%)		9 (3.5%)	7 (2.7%)	
M stage, n (%)			0.052			0.090
M0	209 (41.3%)	219 (43.3%)		210 (41.5%)	218 (43.1%)	
M1	48 (9.5%)	30 (5.9%)		47 (9.3%)	31 (6.1%)	
Age, median (IQR)	61 (53, 70)	60 (51, 69)	0.366	61.33 ± 11.9	59.93 ± 12.26	0.178

### Functional enrichment

**Figure 3:**
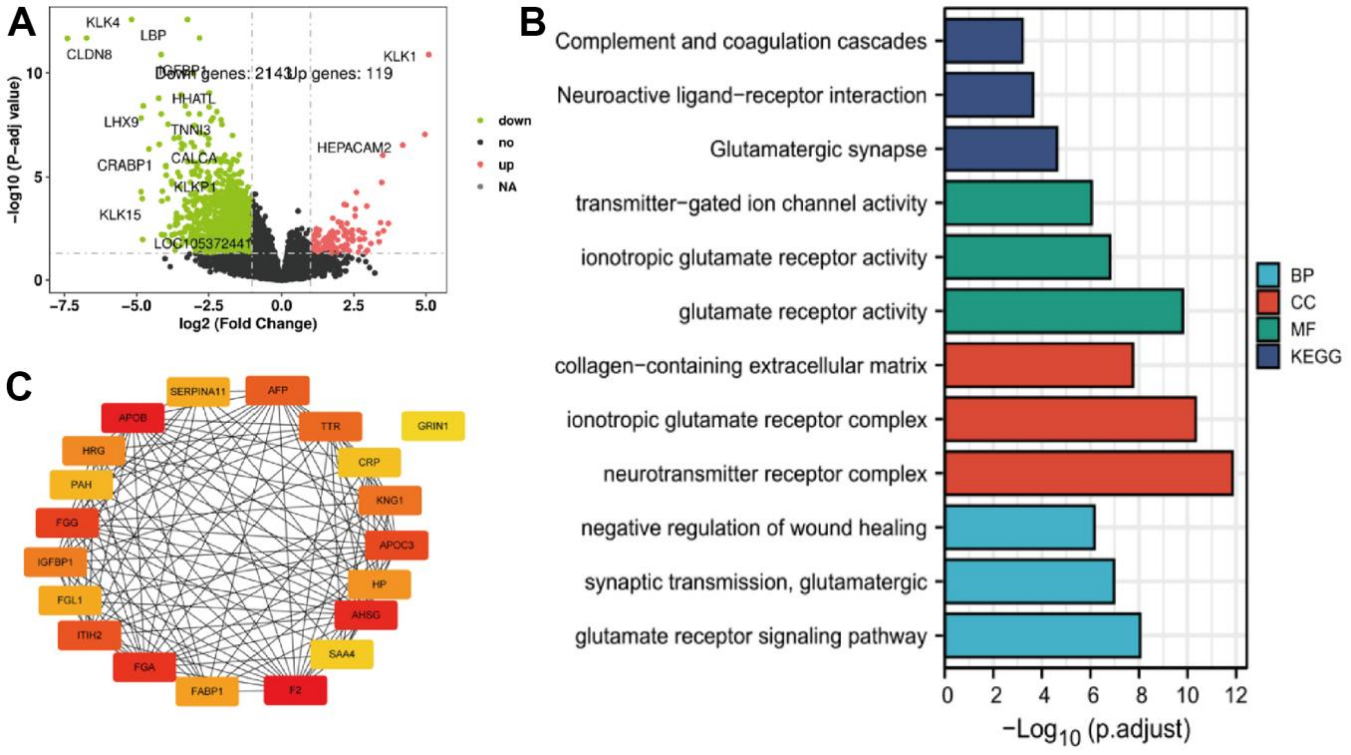
(**A**–**C**) The TRIM26 different expression genes' volcano figure, PPI network, and enrichment function analysis.

Many mRNA were down-regulated in patients who exhibited high expression of TRIM genes, relative to with low expression. A total of 2143 and 119 were down-regulated and up-regulated, respectively for TRIM26 (Figure 3A). In addition, we identified the top 20 hub genes, and used Cytoscape to generate a protein-protein interaction by network (Figure 3B). Results of subsequent GO and KEGG analysis are presented in Figure 3C.

### Prediction of downstream TRIM genes regulating ubiquitination

**Figure 4:**
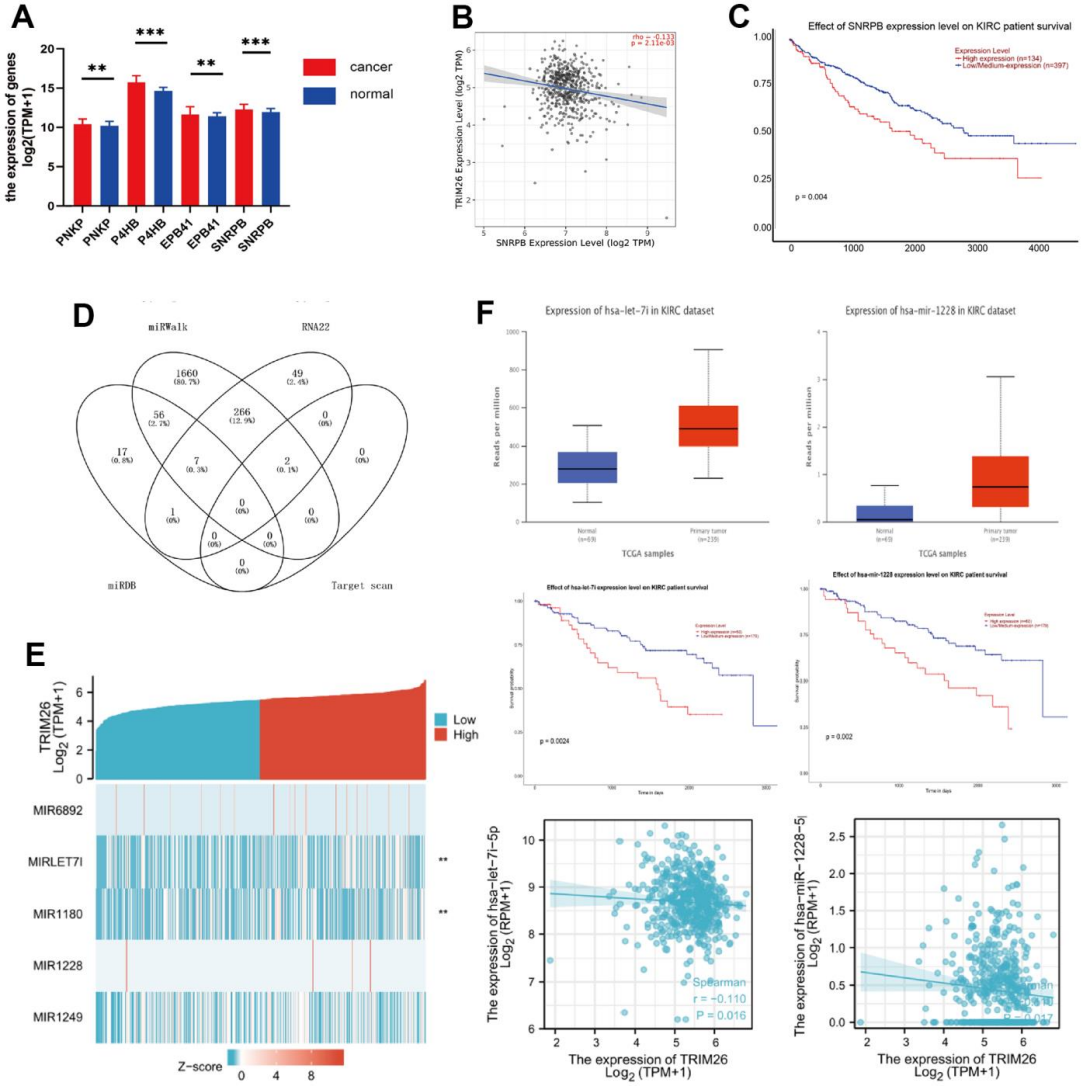
(**A**) The different expression genes of TRIM26 potential downstream; (**B**) The correlation between TRIM26-SNRPB; (**C**) Survival analysis of SNRPB; (**D**) The Venn diagram of miRNAs for TRIM26; (**E**) The correlation between TRIM26 and miRNAs' expression level; (**F**) The expression level, the survival analysis, and the correlation for hsa-let-7i-5p/hsa-miR-1228-5p.

Analysis of iUUCD dataset revealed 24 potential ubiquitination genes downstream of TRIM26, respectively. Next, we applied ubibrowser tool and identified potential ubiquitin genes downstream of TRIM26. We retrieved the top 50 genes, as targets for TRIM26 ubiquitination from the ubibrowser tool, then applied the UALCAN tool to analyze expression levels and survival value of the potential genes in the KIRC dataset (Supplementary Table 1). Results showed that 4 (PNKP, P4HB, EPB41, and SNRPB) downstream of TRIM26 were significantly differentially expressed (P <0.01) and had significant survival value (P <0.01) (Figure 4A). Furthermore, results from TIMER revealed a significant negative correlation between SNRPB/TRIM26 in KIRC patients (Figure 4B, 4C). We speculated that SNRPB, which may play a ubiquitination role in KIRC tissues, may be directly interacting with TRIM26.

### Prediction of upstream miRNAs

We used four miRNA prediction databases to predict potential miRNAs upstream of target TRIM26. We found 81 miRNAs from the miRDB database, as well as 1991, 325, and 2 others from miRWalk, RNA22, and target scan, respectively, for TRIM26(Figure 4D). Analysis of intersection of these miRNAs revealed a total of 323 miRNAs supported by two miRNA databases for TRIM26. Analysis of all potential miRNAs for TRIM26, using UALCAN, revealed significant differential expression of 5 miRNAs (P <0.001, Figure 4E). Moreover, KM curves indicated that they had significant survival value (P<0.05) for TRIM26. We further correlated the 5 miRNAs with levels of TRIM26 expression in KIRC patients and found a significant negative correlation in two miRNAs (hsa-let-7i-5p, and hsa-miR-1228-5p) (P <0.05, Figure 4F).

### Construction and analysis of a risk-score signature

**Figure 5:**
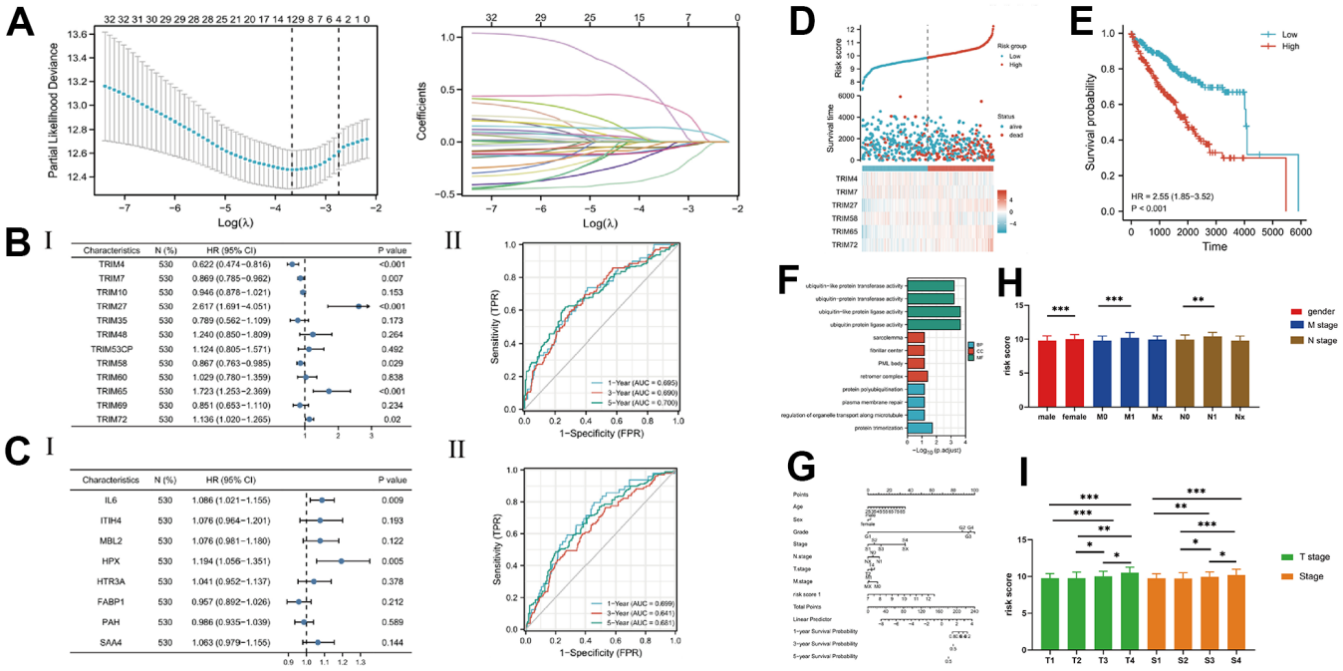
(**A**) The lasso analysis; (**B**, **C**) The Cox analysis and ROC analysis for two risk score; (**D**) The risk score and 6 TRIM genes' expression level; (**E**) The KM analysis of risk score; (**F**) The nomogram of the risk score and other clinical information; (**G**) The enrichment function analysis; (**H**, **I**) The detail expression level of the risk score in different clinical stages.

Analysis of all TRIM genes, using Lasso Regression, revealed 12 significant genes, while cox proportional hazard regression identified 6 (TRIM4, TRIM7, TRIM27, TRIM65, TRIM58, and TRIM72) after the (Figure 5A). Combining TRIM26 with their relevant 20 hub genes resulted in 21 genes. Next, we applied Lasso and cox analyses and found only two genes (IL6, and HPX) left. We compared two risk score prognostic value and found that the TRIM genes’ risk score (6 TRIM genes) had better AUC values for 3-year and 5-year, and selected the TRIM genes as our risk score (Figure 5B, 5C). The risk score = (-0.474815186*Exp TRIM4) + (-0.140412154*Exp TRIM7) + (0.962028624*Exp TRIM27) + (-0.142716302*Exp TRIM58) + (0.544066958*Exp TRIM65) + (0.12751332*Exp TRIM72). We analyzed the risk score by generating KM curves and a nomogram prediction model (Figure 5D, 5E), then performed functional enrichment, and correlation of clinical information. Results showed that the KIRC patients with higher risk scores were associated with worse clinical information (Figure 5G–5I), such as poor pathological stage, and short survival times. Furthermore, results from functional enrichment showed that risk scores were correlated with ubiquitin-protein activity (Figure 5F).

### Analysis of tumor-infiltrating immune cells

**Figure 6:**
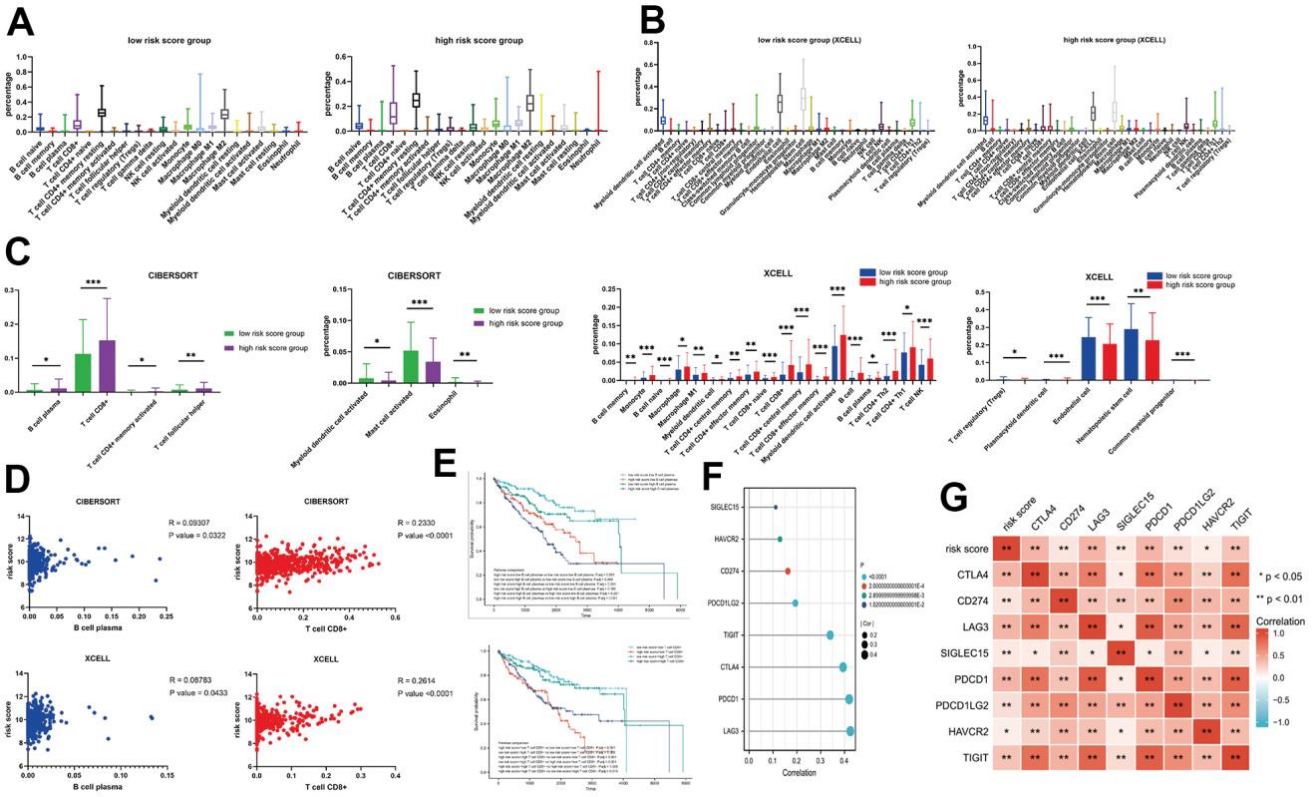
(**A**) The immune components between different risk score groups in the CIBERSORT; (**B**) The immune components between different risk score groups in the XCELL; (**C**) The different immune components for CIBERSORT and XCELL; (**D**) The correlation between the T cell CD8+ / B cell plasma and risk score; (**E**) The KM analysis for the risk score combined with T cell CD8+ / B cell plasma; (**F**, **G**) The interaction plots for immune markers and risk score.

Results from TIMER showed the function for all the TCGA-KIRC patients, including more than 100 immune cell component or fibroblast score. Our focus was on the CIBERSORT dataset, which comprised 22 immune cell components, and XCELL dataset that had 35 immune cell components (Figure 6A, 6B). Results for both risk score groups indicated that patients with high-risk scores were associated with an increase and decrease in 4 and 3 cell components, respectively, in the CIBERSORT (P <0.05). With regards to XCELL, patients with high-risk scores exhibited a significant increase and decrease in 18 and 5 cell components, respectively (P <0.05) (Figure 6C). Analysis of the intersection between CIBERSORT and XCELL results revealed that patients with high-risk scores exhibited an increase in 2 cell components (B cell plasma, and T cell CD8+). Analysis of the correlation and survival value between these cells showed that risk scores were strongly associated with expression levels of B cell plasma and T cell CD8+ (Figure 6D), while survival analysis showed that patients with high risk scores, who had high expression of B cell plasma, exhibited the worst survival times (P < 0.001) (Figure 6E). However, patients with high-risk scores, combined with low expression of T cell CD8+, had worst survival times (P < 0.001). A comparison between risk scores with 8 immune-related genes (LAG3, PDCD1, CTLA4, TIGIT, PDCD1LG2, CD274, HAVCR2, SIGLEC15), revealed a significant correlation among them (P < 0.05). Notably, the top 3 genes with the strongest correlation were LAG3, PDCD1, and CTLA4 (P < 0.001) (Figure 6F, 6G).

## DISCUSSION

TRIM26 is a member of TRIM family genes which includes three zinc-binding domains, a RING, a B-box type 1 and a B-box type 2, and a coiled-coil region. The protein localizes to cytoplasmic bodies [23]. The gene localizes to the major hist-compatibility complex (MHC) class I region on chromosome 6. There were a few types of research that focused on the function and mechanism of TRIM26 for patients with cancer. it was reported that inhibition of TRIM26 could inhibit the non-small lung cells grow [24]. And knockdown of TRIM26 could inhibit the proliferation, migration, and invasion of bladder cancer cells [25]. On the other hand, over-expression of TRIM26 could suppress the proliferation and metastasis of papillary thyroid carcinoma cells [26]. However, there was few research that tried to explain the value of TRIM26 for the KIRC patients.

Our results indicated that TRIM26 has significant value in survival of KIRC patients. Firstly, the gene was significantly down-regulated in KIRC tissues, while KIRC patients with low expression exhibiting worse survival times, as well as poor clinical outcomes, such as poor pathological stage. Further analysis revealed that SNRPB was the potential downstream gene for TRIM26. In fact, up-regulation of TRIM26 resulted in significant down-regulation of SNRPB in tissues, possibly due to ubiquitination. The SNRPB is encoded by several nuclear proteins that are found in common among U1, U2, U4/U6, and U5 small ribonucleoprotein particles (snRNPs). These snRNPs are involved in pre-mRNA splicing, and the encoded protein may also play a role in pre-mRNA splicing or snRNP structure [27]. Because the SNRPB might play the function of mRNA splicing, a few studies had found the expression of SNRPB had a relation with different survival results of several cancers (for example liver cancer [28] and cervical cancer [29]). we believed TRIM26, which may regulate the SNRPB, influenced the KIRC patients’ survival results.

We found two potential miRNAs for the TRIM26, namely hsa-let-7i-5p, and hsa-miR-1228-5p. two miRNAs had significant expression level, survival value in the KIRC tissue, and two miRNAs still had a negative correlation with TRIM26. hsa-let-7i-5p [30] and hsa-miR-1228-5p [31] are short (20-24 nt) non-coding RNAs that are involved in post-transcriptional regulation of gene expression by affecting both the stability and translation of mRNAs. the hsa-let-7i-5p/hsa-miR-1228-5p-TRIM26-SNRPB was a potential mechanism axis that may play a role on the KIRC cells.

Furthermore, we constructed a survival-related risk score signature for identification of more significant indices in KIRC patients. Firstly, we used all the TRIM genes to construct the risk score; option two, we used the TRIM26 and their strong correlation genes. Secondly, we applied Lasso and cox survival regression analyses to identify potential risk scores and found that risk scores from option one had a better AUC value following time-dependent ROC analysis. Therefore, we adopted option one for construction of the risk score, and incorporated TRIM4, TRIM7, TRIM27, TRIM65, TRIM58, and TRIM72. Results from several methods indicated that this new risk score signature had satisfactory survival rates for patients, with a high-risk-score KIRC patients exhibiting significantly worse survival time.

Furthermore, results from analysis of immune micro-environment showed that KIRC patients with high-risk score had a bigger percentage of B cell plasma and T cell CD8+, as well as up-regulation of immune-related genes, like LAG3, PDCD1, and CTLA4. From the immune micro-environment angle of view, KIRC patients with higher-risk score, who had a more percentage of B cell and CD8+ T cells, may have a better effect of immunotherapy. And anti-CTLA4 drugs could be considered in the earlier time for KIRC patients with a higher risk score.

## CONCLUSIONS

Our results revealed that TRIM26 exhibited significant differential expression and survival value in KIRC patients. Further analysis indicated that SNRPB might be the potential downstream for TRIM26, while hsa-let-7i-5p, or hsa-miR-1228-5p were miRNAs upstream of TRIM26. Ultimately, we constructed a survival-related risk score, comprising TRIM4, TRIM7, TRIM27, TRIM65, TRIM58, and TRIM72.

## Supplementary Material

Supplementary Table 1
