# Supplementary material for: Comprehensive analysis of expression profiles and prognosis of TRIM genes in human kidney clear cell carcinoma
Source: Aging (Albany NY). 2022 May 26;14(10):4606–17. doi: 10.18632/aging.204102 (PMC9186766; doi:10.18632/aging.204102)
Supplement: Supplementary Table 1 [file aging-14-204102-s001.pdf]

SUPPLEMENTARY TABLE

Supplementary Table 1. Prediction of downstream TRIM26 regulation ubiquitination.

From the iUUCD results (n=24)	From the ubibrowser tool (n=50)
CAND1, MEPCE, MNAT1, USP36, UBE2D1, UBE2D3, OTUB2, SOX2, SRRM2, MAGEA6, CEBPD, SUMO2, USP5, TRIM41, USP39, PHF7, RNF126, UBC, RABEP1, RNF10, SON, PNKP, RNF2, UBE2D2	PRKD1, DDX54, SMARCA5, DDX47, RPS6KA3, DDX52, HMGB1, YBX3, ARAF, P4HB, A2M, ERCC3, HNRNPD, STRN, SRRM1, ARFGEF1, LRP1, ZMYM2, POLR3F, BCR, EPB41, RPLP0, GPN1, UBR1, TSR1, CWC22, AP1B1, TP53RK, THOC2, LSM1, IFIT2, SNRPB, BAG3, NF2, MARK2, TUBB4B, CNBP, CDC5L, MED12, ABCF3, FTSJ3, SEPTIN2, POLA1, BAD, SNCA POLR2D, HNRNPA2B1, PARD3, STRN3, EXPSC10
